# Comparison of preoperative education by artificial intelligence versus traditional physicians in perioperative management of urolithiasis surgery: a prospective single-blind randomized controlled trial conducted in China

**DOI:** 10.3389/fmed.2025.1543630

**Published:** 2025-06-25

**Authors:** Hui Zhang, Xianjing Wang, Hongyan Luo, Weiyong Zeng, Xuwei Hong, Jiasheng Feng, Guangming Lu, Yongquan Su, Wenting Tong, Yang Xiao

**Affiliations:** ^1^Department of Urology, Dongguan Hospital, Guangzhou University of Chinese Medicine, Dongguan, China; ^2^Department of Experimental Medicine, University of Rome Tor Vergata, Rome, Italy; ^3^Department of Rehabilitation and Elderly Care, Gannan Healthcare Vocational College, Ganzhou, Jiangxi, China; ^4^Department of Respiratory Medicine, Dongguan Hospital of Guangzhou University of Chinese Medicine, Dongguan, China; ^5^Department of Pharmacy, Gannan Healthcare Vocational College, Ganzhou, Jiangxi, China

**Keywords:** preoperative anxiety, urolithiasis, patient education, artificial intelligence, ChatGPT, Ernie Bot

## Abstract

**Background:**

Preoperative anxiety is common in patients awaiting urolithiasis surgery. Although adequate preoperative education can reduce anxiety and improve outcomes, time and resource constraints often limit the depth and personalization of such interventions. Recent advances in AI, particularly large language models like GPT-4o and Ernie Bot, offer potential tools to supplement traditional education. However, their comparative effectiveness remains unclear.

**Purpose:**

This randomized controlled trial compared the effectiveness of AI-based preoperative education (ChatGPT, Ernie Bot) to that provided by attending urologists in reducing anxiety, enhancing satisfaction, and improving information quality.

**Methods:**

Seventy-five adult patients scheduled for urolithiasis surgery were randomly assigned (1:1:1) to ChatGPT, Ernie Bot, or urologist-led education. All received a standardized consultation 12 h preoperatively. The AI groups then had a 30 min Q&A with their assigned AI, and the physician group received an additional face-to-face session. Anxiety was measured via the State–Trait Anxiety Inventory (STAI) at baseline, immediately post-intervention, 3 h pre-op, and 3 h post-op. Secondary outcomes included patient satisfaction, query count, evaluation of information quality (safety, accuracy, empathy, readability, detail), and postoperative (3 h) pain (VAS).

**Results:**

All participants completed the study. Anxiety decreased significantly after intervention in all groups (*p* < 0.05). The physician group achieved the greatest anxiety reduction, followed by ChatGPT and then Ernie Bot. At 3 h pre-op, physician and ChatGPT groups maintained lower anxiety, while Ernie Bot showed a non-significant rebound. Postoperatively, all groups had significantly lower anxiety than baseline. ChatGPT excelled in empathy, readability, and detail, and elicited twice as many patient questions. Satisfaction was high in both the physician and ChatGPT groups. Ernie Bot’s responses, though safe and accurate, were conservative and less detailed, leading to fewer inquiries and lower satisfaction. Postoperative pain was lowest in the physician group, followed by ChatGPT (*p* < 0.05).

**Conclusion:**

AI-assisted education, particularly via ChatGPT, can effectively reduce preoperative anxiety and improve patient engagement, though not to the level of physician-led education. Ernie Bot showed modest benefits. Further refinement of AI may enhance its role as a supplemental educational tool.

## Introduction

Preoperative anxiety refers to the unease and fear patients experience while awaiting surgery due to uncertainty (1). This phenomenon is highly prevalent in clinical practice (2–5). Studies indicate that the global incidence of preoperative anxiety may be as high as 60 to 92% (6, 7). Among patients undergoing urological surgery, preoperative anxiety tends to be more pronounced due to the intimate nature of the surgical site and the involvement of sensitive topics, which may significantly impact postoperative recovery and patient satisfaction (8, 9). Such anxiety often arises from uncertainty surrounding the surgical procedure itself, the anesthesia process, potential complications, and postoperative recovery (4, 10, 11). Beyond psychological distress, anxiety influences endocrine and nervous system responses (12), particularly the sympathetic nervous system and the hypothalamic–pituitary–adrenal (HPA) axis (13), thereby increasing pain sensitivity after surgery, prolonging hospitalization, elevating the risk of postoperative complications, and potentially affecting surgical outcomes (10). It can also induce tachycardia, elevated blood pressure, sweating, abnormal body temperature, excessive worry, tension, and even increased aggression (14, 15), which compromise the stability of anesthesia induction and maintenance, increase anesthetic requirements, and contribute to autonomic instability (16). Therefore, identifying effective and easy-to-implement preoperative interventions to reduce patient anxiety is of great significance for improving perioperative outcomes.

Preoperative education plays a pivotal role in reducing anxiety and improving perioperative outcomes among patients undergoing urological surgery (17). However, in real-world clinical practice, insufficient preoperative education and limited physician-patient communication frequently lead to heightened anxiety, adversely affecting not only postoperative recovery but also the establishment of trust between patients and healthcare providers (9, 18). Studies have shown that inadequate preoperative education is associated with higher levels of anxiety, reduced patient understanding, and increased risks of postoperative nausea, vomiting, and infection (8, 15, 16). Conversely, comprehensive and clear preoperative education significantly reduces patient anxiety and fear, thereby improving clinical outcomes and patient satisfaction (10, 19, 20). Despite this evidence, the implementation of high-quality education remains challenging. Clinicians often face constraints in time, energy, and workforce, making it difficult to deliver sufficient, individualized, and sustainable patient education in the perioperative period (18). These challenges manifest not only as a lack of timely and repeatable information exchange, but also as communication barriers and inflexible formats that may discourage patients from asking questions or fully understanding the information provided (21, 22). As a result, there is a pressing need to explore innovative, scalable, and patient-centered educational solutions to address these shortcomings in contemporary clinical environments.

The advent of artificial intelligence (AI) technologies presents new opportunities to address these limitations. Internationally, large language models (LLMs) such as GPT-4o (an OpenAI model released on May 14, 2024) (23) and Ernie Bot 3.5 (a widely adopted AI model developed by Baidu and released on March 20, 2023) (24) have garnered significant attention due to their advanced natural language processing capabilities and interactive features (23, 25). Existing literature suggests that AI tools can enhance the accessibility, personalization, and quality of medical consultations and education, especially by enabling repeated, on-demand information access and improving patient engagement (24–27). Preliminary studies have shown that LLM-based platforms may be effective in simulating human consultation, answering patient questions, and even improving comprehension and satisfaction in some healthcare settings (28). However, robust, comparative, and outcome-based evidence on the role of AI—particularly large language models—in reducing preoperative anxiety and improving perioperative experiences is still limited (18). In the field of urology, such evidence is particularly lacking, with most existing studies focusing on general medical education or technical performance rather than emotional and cognitive outcomes in surgical patients.

Given the limited availability of physician time and resources in clinical practice, alongside patients’ growing demand for comprehensive, accessible, and personalized medical information, this study aims to systematically compare the effectiveness of three educational approaches: AI-based education using GPT-4o, Ernie Bot, and conventional education provided by attending urologist in reducing preoperative anxiety preoperative anxiety in patients undergoing urological stone surgery. To our knowledge, this is the first prospective randomized controlled trial to directly evaluate the comparative efficacy of multiple LLM-based education tools versus human physician education in a surgical setting.

We anticipate that the findings will offer robust evidence to support the integration of AI-assisted patient education into routine clinical workflows. Moreover, these results may inform future strategies to reduce disparities in healthcare delivery, optimize perioperative management, and bridge the gap between patients’ information needs and the limited availability of healthcare professionals.

## Methods

### Study design and participants

This single-center, prospective, randomized controlled trial was conducted from September 4, 2024, to November 30, 2024, at the Department of Urology, Dongguan Hospital of Guangzhou University of Chinese Medicine, located in Dongguan, Guangdong, China. We recruited consecutive patients hospitalized for urological stone surgery. After obtaining written informed consent, eligible patients were randomized equally into three groups (ChatGPT, Ernie Bot, traditional physician education) using a computer-generated randomization list (Python random module), prepared by an independent statistician not involved in recruitment or assessment. Allocation concealment was ensured using sequentially numbered, sealed, opaque envelopes prepared in advance. Outcome assessors were blinded to group assignments to minimize observer bias.

### Inclusion criteria

1. Adults aged ≥18 years.
2. No previous surgical history.
3. Scheduled for urological stone surgery.
4. Able and willing to receive AI-based educational interventions.
5. Adequate language comprehension and expression skills to complete questionnaires and provide feedback.
6. Understanding of the study and provision of written informed consent.

### Exclusion criteria

1. Cognitive impairment or severe mental disorders (e.g., active major depressive or severe anxiety disorders) that may affect comprehension or questionnaire completion.
2. Prior participation in similar AI-based or preoperative urological stone surgery education studies.
3. Strong prejudice against or refusal to use AI technology.
4. Emergency surgeries that preclude completion of preoperative education.
5. Severe comorbidities (e.g., uncontrolled heart disease, renal failure) that could affect study outcomes or increase health risks.
6. Severe language barriers preventing comprehension or feedback.

### Withdrawal criteria

1. Noncompliance with the study schedule, questionnaires, or educational procedures.
2. Occurrence of adverse events (AEs), such as inappropriate or misleading information, causing increased anxiety or fear and necessitating withdrawal for medical intervention.
3. Participant-initiated withdrawal at any time.
4. Loss to follow-up, where participants cannot be contacted or fail to attend scheduled visits, resulting in incomplete data.
5. Use of non-approved external information sources contrary to study protocols.
6. Significant changes in the participant’s health status (e.g., diagnosis of a new severe illness or exacerbation of existing conditions) that hinder continued participation.

### Interventions and control conditions

In this study, the physicians referred to as “attending urologists” were board-certified clinicians who had completed a master’s degree in clinical medicine, passed the national standardized residency training program, and accrued at least 2 years of independent clinical practice, fulfilling all requirements for the Attending Physician title in China. They were responsible for delivering standardized consultations, conducting Q&A sessions in the physician group.

All participants received a standardized consultation 12 h before surgery, delivered by an attending urologist to ensure uniform baseline education. Following this consultation:

**ChatGPT Group and Ernie Bot Group:** Participants engaged in open-ended text or voice-based interactions with their assigned AI (ChatGPT or Ernie Bot), without restrictions on the number of questions or depth of discussion.
**Traditional Physician Education Group:** After the standard consultation, participants received an additional 30 min face-to-face Q&A session and supplemental explanations from a attending urologist.

All interactions were recorded (audio or text) with participant consent. These records were later reviewed by the board-certified chief urologist blinded to group assignments.

### Validation of AI responses

To evaluate the accuracy and readability of AI-generated responses, we implemented a structured expert review method. Two board-certified chief urologists independently reviewed and scored all AI responses using a 10-point scale. For accuracy, scoring criteria included:

- Adherence to established clinical standards and current guidelines.
- Absence of medical misinformation or misleading content.
- Overall safety and clinical appropriateness.

Readability was also scored on a 10-point scale, assessing:

- Clarity and avoidance of excessive medical jargon.
- Ease of understanding for non-medical audiences.
- Relevance and educational value for the preoperative context.

Experts were instructed to assess from the perspective of a patient’s comprehension needs. Inter-rater consistency was confirmed through preliminary calibration, and discrepancies were resolved by consensus. This approach was informed by methodologies used in previous studies evaluating LLMs in medical education and clinical decision support.

### Outcome measures

#### Baseline data

Age, sex, height, weight, education level, relevant comorbidities (hypertension, diabetes, chronic kidney disease), ASA classification, stone location, and surgical type.

#### Primary outcome

State–Trait Anxiety Inventory (STAI) scores at four time points: pre-intervention (12 h preoperatively), immediately post-intervention, 3 h preoperatively, and 3 h postoperatively, using the STAI short form (29). The cut-off values were 9.5 for state anxiety and 13.5 for trait anxiety.

#### Secondary outcomes

Number of participant questions asked.

Education quality metrics: Safety, Empathy, Readability, Detail Level, Participant Understanding, Error Count, Question Count, Participant Satisfaction.

Postoperative pain at 3 h measured by Visual Analog Scale (VAS).

All adverse events were recorded and addressed. Safety, accuracy, empathy, readability, informational detail, postoperative pain (VAS), and satisfaction with preoperative education were rated on a 10-point scale.

#### Sample size calculation

Based on pilot data and assuming a moderate-to-large effect size (*Δ* = 0.8), with power (1−*β*) = 0.8 and *α* = 0.05, the sample size per group was calculated using:

$$n=\frac{2(\sigma^{2}){\left(z_{\alpha ∕2}+\mathrm{z\beta}\right)}^{2}}{\Delta^{2}}$$

This yielded approximately 25 participants per group, totaling 75 participants.

### Statistical methods

A preliminary analysis indicated non-normal data distributions. Thus, nonparametric methods were used for both between-group and within-group comparisons. Data were grouped by intervention type (ChatGPT, Ernie Bot, Urologist) to evaluate differential effects. Baseline differences were assessed using the Kruskal-Wallis H, ANOVA, and Chi Square test. If significant differences emerged, pairwise comparisons were conducted using Mann–Whitney U tests with Bonferroni correction. Cohen’s d was calculated to estimate effect sizes. Statistical significance was set at *p* < 0.05 (two-sided). All analyses were performed in Python, employing SciPy for nonparametric tests, statsmodels for multiple comparisons, and NumPy and Pandas for data management and effect size calculations. All results, including non-significant findings, are comprehensively reported in tables.

### Ethical considerations

This study protocol was approved by the Institutional Review Board of the Dongguan Hospital of Guangzhou University of Chinese Medicine (No. 2024-70). The study adhered to the Declaration of Helsinki and ICH-GCP guidelines. All participants provided written informed consent after a full explanation of the study.

## Results

A total of 75 participants were recruited (25 per group). No participants withdrew, were lost to follow-up, or discontinued participation. Demographic and baseline characteristics are shown in Table 1.

**Table 1:** Comparison of participant characteristics among groups.

Category	ChatGPT	Ernie_Bot	Urologist	Statistic-value	Effect size	*p*-value
	(*n* = 25)	(*n* = 25)	(*n* = 25)			
Age	49.68 ± 13.80	47.96 ± 12.20	48.80 ± 13.71	0.1025*	−0.003	0.900
BMI	22.94 (21.50, 25.32)	22.77 (21.22, 25.12)	22.91 (21.70, 25.08)	0.142#	−0.026	0.932
Gender				0.50ξ	0.058	0.78
Male	18/25 (72%)	18/25 (72%)	16/25 (64%)			
Female	7/25 (28%)	7/25 (28%)	9/25 (36%)			
Ethnic				2.03ξ	0.116	0.36
Hans	25/25 (100%)	24/25 (96%)	25/25 (100%)			
Others	0/25 (0%)	1/25 (4%)	0/25 (0%)			
Occupation				11.81ξ	0.281	0.46
Driver	0/25 (0%)	0/25 (0%)	1/25 (4%)			
Farmer	0/25 (0%)	1/25 (4%)	0/25 (0%)			
Retiree	7/25 (28%)	3/25 (12%)	7/25 (28%)			
Server	0/25 (0%)	0/25 (0%)	1/25 (4%)			
Staff	12/25 (48%)	9/25 (36%)	6/25 (24%)			
Technician	0/25 (0%)	2/25 (8%)	2/25 (8%)			
Worker	6/25 (24%)	10/40 (40%)	8/25 (32%)			
Income (China Yuan)				0.87ξ	0.076	0.93
<5 k	18/25 (72%)	17/25 (68%)	18/25 (72%)			
5 k–10 k	6/25 (24%)	7/25 (28%)	5/25 (20%)			
10 k–20 k	1/25 (4%)	1/25 (4%)	2/25 (8%)			
Diagnosis				3.38ξ	0.15	0.76
Ureter	18/25 (72%)	15/25 (60%)	15/25 (60%)			
Kidney	6/25 (24%)	6/25 (24%)	6/25 (24%)			
Bladder	0/25 (0%)	1/25 (4%)	2/25 (8%)			
Urethra	1/25 (4%)	3/25 (12%)	2/25 (8%)			
Anatomic location of stones				6.61ξ	0.210	0.76
Cystolithotripsy	0/25 (0%)	3/25 (12%)	2/25 (8%)			
Laparoscopic Ureterolithotomy	1/25 (4%)	1/25 (4%)	0/25 (0%)			
Percutaneous Nephrolithotomy	2/25 (8%)	0/25 (0%)	2/25 (8%)			
Retrograde Intrarenal Surgery	5/25 (20%)	6/25 (24%)	5/25 (20%)			
Ureteroscopy-Lithotripsy	16/25 (64%)	14/25 (56%)	14/25 (56%)			
Urethral Stone Lithotripsy	1/25 (4%)	1/25 (4%)	2/25 (8%)			

*: ANOVA, #: Kruskal-Wallis, ξ: Chi Square.

Compared with baseline, anxiety scores declined significantly in all groups immediately after the intervention (*p* < 0.05) (Table 2). At 3 h preoperatively, scores slightly rebounded from immediate post-intervention levels. Specifically, in the ChatGPT and Urologist groups, anxiety levels at 3 h preoperatively remained significantly lower than at baseline (*p* < 0.05), whereas in the Ernie Bot group, anxiety rose slightly, though not significantly (*p* > 0.05) (Table 3). By 3 h postoperatively, anxiety levels in all groups were significantly lower than at baseline (*p* < 0.05) (Table 4).

**Table 2:** Group comparisons at baseline and immediately after preoperative education (12 hours preoperatively).

Group	Preoperative (12 h before surgery)												Intervention results
	STAI_SA	STAI_TA	ASA	Safety	Empathy	Readability	Detail_Level	Participant_Understanding	Error_Count	Question_Count	Participant_Satisfaction	AEs	STAI_SA
ChatGPT	14 (12, 15)	10 (8, 11)	1 (1, 1)	0 (0, 0)	8 (7, 9)	6 (5, 7)	8 (7, 8)	8 (7, 8)	3	23 (22, 28)	8 (7, 8)	0	10 (9, 11)*
Ernie_Bot	13 (12, 14)	10 (9, 11)	1 (1, 1)	0 (0, 0)	4 (3, 4)	4 (4, 5)	5 (5, 6)	4 (3, 5)	0	10 (9, 11)	4 (3, 4)	0	12 (10, 13)*
Urologist	14 (12, 14)	11 (10, 12)	1 (1, 1)	0 (0, 0)	6 (5, 7)	6 (5, 6)	6 (6, 7)	7 (6, 8)	0	11 (10, 11)	7 (6, 8)	0	9 (7, 9)*

STAI_SA: State–Trait Anxiety Inventory, State Anxiety; STAI_TA: State–Trait Anxiety Inventory, Trait Anxiety; ASA: American Society of Anesthesiologists Physical Status Classification; AEs: Adverse Events; VAS: Visual Analog Scale.

**Table 3:** Group comparisons of key outcomes at 3 hours preoperatively and 3 hours postoperatively.

Group	Preoperative (3 h before surgery)		Postoperative (3 h after)	
	STAI_SA	AEs	STAI_SA	VAS
ChatGPT	12 (10, 13)*	0	8 (7, 9)*	2 (2, 3)
Ernie_Bot	14 (13, 15)	0	12 (11, 13)*	3 (3, 4)
Urologist	10 (8, 10)*	0	6 (5, 7)*	2 (2, 2)

STAI_SA: State–Trait Anxiety Inventory, State Anxiety; STAI_TA: State–Trait Anxiety Inventory, Trait Anxiety; ASA: American Society of Anesthesiologists Physical Status Classification; AEs: Adverse Events; VAS: Visual Analog Scale. * Indicates significant within-group reduction compared with baseline (*p* < 0.05).

**Table 4:** Comparison of outcomes across groups at different time points.

Time point	Variable	Group 1	Group 2	Effect size (Cohen’s d)	u-statistic	*p*-value
Preoperative (12 h before surgery)	STAI_SA	ChatGPT_4o	Ernie_Bot	0.326899	372	0.73
			Urologist	0.0881068	330	1
		Ernie_Bot	Urologist	−0.241897	268.5	1
	STAI_TA	ChatGPT_4o	Ernie_Bot	−0.136452	286.5	1
			Urologist	−0.835185	180	0.06
		Ernie_Bot	Urologist	−0.722078	193	0.05
	ASA	ChatGPT_4o	Ernie_Bot	0	312.5	1
			Urologist	−0.243057	287.5	1
		Ernie_Bot	Urologist	−0.243057	287.5	1
	Safety	ChatGPT_4o	Ernie_Bot	0.5116817	350	0.24
			Urologist	0.5116817	350	0.24
		Ernie_Bot	Urologist	0	312.5	1
	Empathy	ChatGPT_4o	Ernie_Bot	3.8719	625	<0.001
			Urologist	1.9348303	557.5	<0.001
		Ernie_Bot	Urologist	−2.308298	36	<0.001
	Readability	ChatGPT_4o	Ernie_Bot	2.3590713	583	<0.001
			Urologist	0.7680909	425.5	0.05
		Ernie_Bot	Urologist	−2.124876	66	<0.001
	Detail_Level	ChatGPT_4o	Ernie_Bot	3.7724943	625	<0.001
			Urologist	2.2263901	577	<0.001
		Ernie_Bot	Urologist	−1.961161	78	<0.001
	Patient_Understanding	ChatGPT_4o	Ernie_Bot	4.8989795	625	<0.001
			Urologist	0.9968641	458	<0.001
		Ernie_Bot	Urologist	−3.78168	0	<0.001
	Error_Count	ChatGPT_4o	Ernie_Bot	0.5116817	350	0.24
			Urologist	0.5116817	350	0.24
		Ernie_Bot	Urologist	0	312.5	1
	Question_Count	ChatGPT_4o	Ernie_Bot	6.6939442	625	<0.001
			Urologist	6.3368084	625	<0.001
		Ernie_Bot	Urologist	−1.007597	165.5	<0.001
	Satisfaction	ChatGPT_4o	Ernie_Bot	3.6752524	619	<0.001
			Urologist	0.757622	439.5	0.03
		Ernie_Bot	Urologist	−2.859167	16.5	<0.001
	AEs	ChatGPT_4o	Ernie_Bot	312.5	1	1
			Urologist	312.5	1	1
		Ernie_Bot	Urologist	312.5	1	1
Intervention results	STAI_SA	ChatGPT_4o	Ernie_Bot	−1.111039	150	<0.001
			Urologist	1.2939933	502	<0.001
		Ernie_Bot	Urologist	2.4654674	605	<0.001
Preoperative (3 h before surgery)	STAI_SA	ChatGPT_4o	Ernie_Bot	−1.6924	75.5	<0.001
			Urologist	1.5394943	528.5	<0.001
		Ernie_Bot	Urologist	3.5714286	624.5	<0.001
	AEs	ChatGPT_4o	Ernie_Bot	−0.282843	300	1
			Urologist	0	312.5	1
		Ernie_Bot	Urologist	0.2828427	325	1
Postoperative (3 h after)	STAI_SA	ChatGPT_4o	Ernie_Bot	−2.121088	40	<0.001
			Urologist	1.3122657	505	<0.001
		Ernie_Bot	Urologist	4.4375903	625	<0.001
	VAS	ChatGPT_4o	Ernie_Bot	−2.27684	56	<0.001
			Urologist	0.8640988	400	0.01
		Ernie_Bot	Urologist	3.9259818	625	<0.001

STAI_SA: State–Trait Anxiety Inventory, State Anxiety; STAI_TA: State–Trait Anxiety Inventory, Trait Anxiety; ASA: American Society of Anesthesiologists Physical Status Classification; AEs: Adverse Events; VAS: Visual Analog Scale.

Between-group comparisons indicated that immediately post-intervention (12 h preoperatively), the Urologist group achieved the greatest reduction in anxiety, followed by the ChatGPT group, with statistically significant differences among groups (*p* < 0.05). These differences persisted into the postoperative period, where the ChatGPT and Urologist groups maintained significantly lower anxiety levels (*p* < 0.05).

No significant differences were observed in safety (*p* > 0.05). Empathy scores differed significantly among the three groups (*p* < 0.05), and the Ernie Bot group exhibited lower readability compared to the ChatGPT and Urologist groups (*p* < 0.05). Detailedness of responses and participant understanding also differed significantly among groups (*p* < 0.05). The ChatGPT group had three identified errors, while the Urologist and Ernie Bot groups had none.

Regarding the number of questions asked, participants interacting with ChatGPT posed significantly more questions than those in the other two groups (*p* < 0.05). To better understand the nature and content of patient engagement, we conducted a thematic analysis of all questions raised during the preoperative education sessions. Although the timing of questioning occurred exclusively before surgery, the topics spanned the entire perioperative spectrum, including intraoperative and postoperative concerns.

These questions were categorized into four domains reflecting the scope of patient interests:

Surgical understanding and decision-making: including questions about surgical necessity, procedure options, and physician experience (e.g., “Do I really need this surgery?” “Is it laparoscopic or open?”).
Preoperative logistics and preparation: such as fasting, medication use, and required tests (e.g., “Can I still take my blood pressure meds?” “Should I stop aspirin?”).
Anticipated intraoperative and postoperative issues: including anesthesia, pain, complication risks, recovery duration, and organ function outcomes (e.g., “Will anesthesia be general?” “How long will I need help after surgery?” “Will my kidney be affected?”).
General administrative and psychosocial concerns: such as financial coverage, comorbidities, and access to support networks (e.g., “Does insurance cover most of the cost?” “Is there a patient group on WeChat?”).

These findings reflect the breadth and depth of patient concerns and demonstrate the role of AI and physician-led education in addressing not only procedural but also emotional and social aspects of surgical care. The full classification is presented in Supplementary Table 1.

No adverse events related to the preoperative consultation occurred in any group (*p* > 0.05). Satisfaction was relatively high in both the ChatGPT and Urologist groups, with no significant difference between them (*p* > 0.05).

Postoperative VAS scores revealed statistically significant differences among the three groups, with the Urologist group reporting the lowest pain scores, followed by the ChatGPT group (*p* < 0.05) (Table 4).

## Discussion

This study demonstrates that preoperative education, whether delivered by attending urologists or by AI models (ChatGPT and Ernie Bot), significantly reduces patient anxiety. This anxiolytic effect was observed at all assessed time points—immediately after the intervention, 3 hours before surgery, and 3 hours post-surgery—confirming the durability of the benefit. Between-group comparisons revealed that traditional urologists were most effective in lowering anxiety, followed by ChatGPT, and then Ernie Bot. These findings are consistent with previous research on the efficacy of preoperative education in mitigating anxiety and extend the existing evidence for AI’s potential role in medical education and psychological support (18, 30).

Further analysis showed that ChatGPT excelled in empathy, readability, and the richness of detail in its responses. Participants interacting with ChatGPT asked almost twice as many questions as those in the other groups, suggesting that AI-based interactions may foster greater patient engagement and comprehension of preoperative procedures. These advantages highlight AI’s potential for delivering personalized knowledge and psychological support, thereby enhancing patient satisfaction. In contrast, Ernie Bot’s more cautious, less detailed responses may stem from stricter regulatory compliance and content review mechanisms (31). While such caution may reduce liability risks, it might also limit depth and flexibility in addressing patient concerns.

Despite these strengths, human clinicians remain the most effective educators. Their professional judgement, contextual awareness, and nuanced empathy are difficult to replicate algorithmically. Consequently, further localisation and domain-specific fine-tuning will be needed before AI can fully approximate clinician-delivered counselling.

To safeguard safety and reliability, we validated every AI response through structured expert review by two board-certified chief urologists who used a 10-point scale to rate accuracy, clarity, and patient suitability. Expert review remains the most widely adopted evaluation method when patient-level outcome data are unavailable (26, 32).

Nevertheless, AI-assisted education can serve as a valuable complement to conventional preoperative communication, particularly in addressing workforce shortages, time constraints, and resource limitations in routine clinical practice. AI systems can operate around the clock and respond instantly to patient needs, providing flexible and personalized communication services that enhance patient engagement in medical decision-making, reduce preoperative anxiety, and ultimately improve clinical outcomes.

AI-based education can be integrated into existing clinical workflows. For instance, during hospital admission and preoperative preparation stages, hospitals may provide patients with access to AI interfaces via self-service kiosks or dedicated apps, enabling them to interact anytime and anywhere. This further promotes patients’ right to know and satisfaction. Moreover, standardized educational content provided by AI can help optimize resource allocation by allowing medical staff to focus on more complex issues.

Beyond the perioperative phase, AI technologies can also support postoperative recovery and rehabilitation. By providing clear, accessible guidance on wound care, medication adherence, and activity limitations, AI systems may enhance care continuity and patient compliance. Future research should investigate how these tools can be integrated with existing electronic health systems to facilitate seamless clinical implementation.

Considering these findings in the broader context of global healthcare resource distribution further illuminates the complexity of this issue. Marked inequalities exist worldwide in access to quality medical care and insurance coverage (33, 34). Such imbalances challenge the delivery of adequate, let alone personalized, preoperative education and psychological support in many low-resource areas.

Against this backdrop, AI emerges as a promising tool for bridging gaps in medical education and support systems. AI can operate continuously, delivering consistent, rapid responses to large numbers of patients regardless of geographical constraints. Its scalability and sustainability may enable low-cost coverage in remote regions, addressing critical shortages of healthcare professionals and infrastructure (35).

To unlock AI’s potential in promoting equitable access to medical education and information, concerted efforts are necessary. Technological improvements should focus on enhancing the AI’s adaptability to local contexts and cultural nuances, striving to meet international standards in empathy, openness, and detail. Policy and regulatory frameworks must become more inclusive, reducing regional access barriers and ensuring broader populations benefit from these innovations (26). Ethical and legal guidelines should clarify AI’s role as an adjunctive rather than autonomous decision-maker in clinical practice (26, 36).

## Conclusion

While human physicians remain the gold standard for delivering preoperative education aimed at alleviating patient anxiety, our findings demonstrate that AI models such as ChatGPT and Ernie Bot offer a promising complement. With further refinement, supportive policies, and robust ethical oversight, AI-assisted education could significantly enhance patient understanding and satisfaction while addressing systemic limitations in healthcare delivery. These tools may ultimately contribute to narrowing global disparities in access to quality perioperative care and improving overall surgical outcomes.

## Limitations

The present study has several limitations that should be acknowledged. Although the sample size (*n* = 75; 25 per group) met the statistical power requirements for detecting the primary outcome, it may have been insufficient for detecting more subtle differences in subgroup analyses. Additionally, all participants were classified as ASA I, representing a relatively healthy and low-risk population. This homogeneity may limit the generalizability of our findings to patients with higher surgical risk profiles, such as those classified as ASA II–III. Future research should include a broader range of clinical populations to assess the applicability of AI-assisted education across varying health conditions.

Moreover, the single-center design may introduce regional and institutional bias. Conducting multicenter studies across different geographic locations and healthcare systems will be important to evaluate the external validity and cross-cultural adaptability of our findings. Another limitation lies in the retrospective review of AI-generated content. No real-time safety monitoring mechanism was embedded in the system, which may pose a risk in more complex or high-stakes clinical scenarios. Future implementations should consider integrating real-time oversight and content validation protocols to ensure patient safety.

Despite these limitations, the study provides preliminary evidence supporting the feasibility, safety, and partial efficacy of AI-supported preoperative education in the context of urological surgery.

## Future directions

Building on these findings, future work should:

Increase sample sizes and include moderate- to high-risk patients (ASA II +) to test safety and effectiveness across risk strata.
Conduct multicentre, geographically diverse RCTs to evaluate cross-cultural adaptability and generalisability of AI counselling.
Compare a wider array of AI models and training strategies to identify technical features that optimize psychological and educational outcomes.
Integrate AI chatbots with hospital information systems (HIS/EMR) to study interoperability, workflow efficiency, and data security.
Explore AI applications in postoperative recovery, chronic-disease management, and long-term patient education, extending benefits across the entire continuum of care.

## Data Availability

The raw data supporting the conclusions of this article will be made available by the authors, without undue reservation.
