# Supplementary material for: Comparison of preoperative education by artificial intelligence versus traditional physicians in perioperative management of urolithiasis surgery: a prospective single-blind randomized controlled trial conducted in China
Source: Front Med (Lausanne). 2025 Jun 25;12:1543630. doi: 10.3389/fmed.2025.1543630 (PMC12238029; doi:10.3389/fmed.2025.1543630)
Supplement: Supplementary file 1 [file Table_1.DOCX]

Supplementary table 1. Classification of Patient Questions During Preoperative Educational Interactions with AI Chatbots and Urologists

| **Question Category** | **Representative Examples** |
| --- | --- |
| Surgical Indication and Procedure | “Is this surgery really necessary?” “Will it be laparoscopic or open surgery?” |
| Preparation and Medication Management | “How long should I fast before surgery?” “Should I stop my antihypertensive or anticoagulant medications?” |
| Risks and Complications | “What are the chances of complications like bleeding or infection?” “What are the risks for someone with my condition?” |
| Psychological Readiness and Practical Planning | “Can I take a sleeping pill the night before?” “How long will I be out of work or need help from others?” |
| Anesthesia | “Will I get general or spinal anesthesia?” “Is anesthesia safe if I have diabetes or hypertension?” |
| Surgical Process and Intraoperative Logistics | “How long will the surgery take?” “Can my family wait outside and receive updates during the procedure?” |
| Recovery and Wound Care | “How many days will I stay in the hospital?” “When can I get out of bed or have the dressing changed?” |
| Activity and Diet Restrictions | “When can I shower or drive again?” “Is there anything I shouldn’t eat?” |
| Monitoring for Complications | “What symptoms should I watch out for after surgery?” “When will the catheter or drainage tube be removed?” |
| Long-Term Outcomes | “Will this surgery affect kidney function?” “What is the likelihood of recurrence? Will I need regular follow-ups?” |
| Financial and Insurance Issues | “What is the total cost of the surgery? Is it covered by insurance?” |
| Special Populations and Risk Adjustment | “Is this surgery more risky for someone with heart disease or diabetes?” |
| Social and Psychological Support | “Is there a support group or WeChat community I can join after surgery?” |
